# Molecular Insight into the Response of Lactic Acid Bacteria to Bile Acids

**DOI:** 10.3390/biotech13030029

**Published:** 2024-08-02

**Authors:** Caren N. Moreno, Jorge N. Gomez, María P. Taranto, Ana E. Ledesma, Ana Y. Bustos

**Affiliations:** 1 Centro de Investigación en Biofísica Aplicada y Alimentos (CIBAAL-UNSE-CONICET), RN 9, Km 1125, Santiago del Estero 4206, Argentina; carenmoreno28@gmail.com (C.N.M.); nicolasgoib@gmail.com (J.N.G.); ana1ledesma@yahoo.com.ar (A.E.L.); 2 Centro de Referencia de Lactobacilos (CERELA-CONICET), Chacabuco 145, San Miguel de Tucumán 4000, Argentina; ptaranto@cerela.org.ar; 3 Departamento Académico de Química, Facultad de Ciencias Exactas y Tecnologías, Universidad Nacional de Santiago del Estero, Av. Belgrano Sur 1912, Santiago del Estero 4200, Argentina; 4 Facultad de Agronomía y Agroindustrias, Universidad Nacional de Santiago del Estero. Av. Belgrano Sur 1912, Santiago del Estero 4200, Argentina; 5 Facultad de Humanidades, Ciencias Sociales y de la Salud, Universidad Nacional de Santiago del Estero. Av. Belgrano Sur 1912, Santiago del Estero 4200, Argentina

**Keywords:** bile acids, lactic acid bacteria, microbiota, Raman spectroscopy, scanning electron microscopy, zeta potential

## Abstract

Bile acids (BAs) are the main endogenous modulators of the composition and metabolic activity of the intestinal microbiota. In the present work, the effect of conjugated (glycodeoxycholic, glycocholic, taurodeoxycholic, taurocholic acids) and free BAs [cholic acid (CA) and deoxycholic acid (DCA)] on the survival, biological molecules, and structural and surface properties of two potential probiotic lactic acid bacteria (LAB) was evaluated. For this, viability assays, Raman spectroscopy, scanning electron microscopy (SEM), and zeta potential (ZP) measurements were employed. Our results evidenced that free BAs were more toxic than conjugates, with CA being significantly more harmful than deoxycholic acid (DCA). RAMAN studies show that BAs modify the bands corresponding to proteins, lipids, carbohydrates, and DNA. SEM showed that BAs cause surface distortions with depressions and fold formation, as well as incomplete cell division. DCA was the one that least altered the ZP of bacteria when compared to CA and taurodeoxycholic acid, with gradual changes towards more positive values. In general, the magnitude of these effects was different according to the BA and its concentration, being more evident in the presence of CA, even at low concentrations, which would explain its greater inhibitory effect. This work provides solid evidence on the effects of BAs on LAB that will allow for the development of strategies by which to modulate the composition of the microbiota positively.

## 1. Introduction

Bile acids (BAs), the main constituents of bile, are synthesized in the liver from cholesterol and released in the intestine to facilitate the digestion of dietary lipids. Before secretion, they are conjugated with taurine or glycine to facilitate their solubility. In addition to their digestive function, BAs are important signal molecules involved in glucose and lipid metabolism, inflammation, and intestinal immunity, among other processes [1,2]. 

Once in the gut, the intestinal microbiota strongly modifies the composition of BAs. Reciprocally, BAs are the main endogenous modulators and remodelers of gut microbial communities [3,4]. Among the microbial transformations, BA deconjugation by bacterial bile salt hydrolase (BSH) enzymes is the most important [5]. BA hydrolysis is involved in host energy metabolism, particularly in the regulation of serum cholesterol levels [6,7]. For this reason, BSH activity is considered a probiotic biomarker [8].

Recent studies have highlighted the relationship between gut microbiota and host health [9,10], as well as the alternative method of positively modulating its composition through the consumption of beneficial microorganisms such as lactic acid bacteria (LAB) [11,12]. For this, LAB strains must tolerate the presence of BAs in the gut, which act as biological detergents affecting cellular viability via several mechanisms such as bacterial cell membranes alteration, protein misfolding, oxidative damage to DNA and RNA, intracellular acidification, and dissipation of the proton motive force [13,14,15]. 

The microbial response to the effect of BAs is considered a multifactorial phenomenon that may include innate or intrinsic and adaptive mechanisms that vary among different bacterial species and strains. The former includes structures and metabolic pathways naturally present in the microbial cell that permit tolerance to the stressor, while adaptive responses involve genotypic and phenotypic modifications that arise as a consequence of the exposure of cells to stress and allow microorganisms to survive in its presence [15,16]. The objective of this work was to explore, at a molecular level, the interaction between LAB strains and conjugated and free BAs in concentrations that simulate those present in the intestinal tract. For this purpose, viability assays, Raman spectroscopy, scanning electron microscopy (SEM), and zeta potential (ZP) measurements were used to evaluate the survival, the biological molecules affected, and the structural and surface properties of potential probiotic LAB, respectively. 

Exploring the mechanisms of LAB response to the presence of LAB will allow us to better understand these complicated phenomena and to develop strategies by which to improve the ability of strains to modify the composition of the gut microbiota positively. 

## 2. Materials and Methods

### 2.1. Microorganism and Culture Conditions

*Lactiplantibacillus* (L.) *plantarum* CB2 and *Lentilactobacillus* (L.) *parabuchneri* CB12, previously isolated from artisanal goat’s milk cheeses, were selected for their potential probiotic properties [17]. The strains, stored at −20 °C, were first activated through inoculation at 1% (*v*/*v*) in MRS broth and incubated for 16 h at 37 °C; then, they were subcultured three times in a similar manner before use.

### 2.2. Consequence of Bile Acid Exposure on Lactic Acid Bacteria Survival

The effect of taurocholic (TCA), glycocholic (GCA), taurodeoxycholic (TDCA), glycodeoxycholic (GDCA), cholic (CA), and deoxycholic (DCA) acids on the survival of both strains was evaluated according to Sesin et al. (2023) [17]. For this purpose, the microorganisms were cultured for 16 h at 37 °C in MRS broth in the absence and presence of 0.05, 0.1, 0.25, 0.5, 1, 1.5, and 2 mM CA and DCA and 2.5, 5, and 7.5 mM GCA, TCA, TDCA, or GDCA. The concentrations are in physiological ranges and were selected taking into account the MIC results obtained previously and the CMC of each BA [18]. 

Cell viability was determined by via A_560nm_ measurements after incubation. Percentage survival was calculated using the following formula:

$$\mathrm{Survival}=(\%)=\frac{N_{t}\times 100}{N_{c}},$$

where $N_{c}$ and $N_{t}$ correspond to A_560nm_ measurements of CB2 or CB12 strains in MRS and MRS media with the addition of BAs at different concentrations, respectively.

To compare the effects of BAs on each strain and between strains, statistical analysis was carried out with the Minitab^®^ 19.1 software program version 19.2020.1. For the multiple comparisons test, Fisher’s LSD test was performed with a confidence level of 95%.

### 2.3. Effect of Bile Acids on the Main Biological Macromolecules of a Lactic Acid Bacteria Using Raman Spectroscopy

*L. parabuchneri* CB12 was grown for 16 h at 37 °C in MRS broth in the presence and absence of CA, DCA, or TDCA using the same range of concentrations used in the antimicrobial activity assay. The cells were then centrifuged at 10,000× *g* for 5 min at 4 °C, washed twice with 20 mM sodium phosphate buffer (pH 7.2), and resuspended in the same volume of the buffer. 

Drops containing 10 μL of each bacterial suspension were deposited on a microscope slide. Spectra were acquired using a Confocal Raman Horiba LabRAM HR Evolution (Instituto de Bionanotecnologia del NOA-INBIONATEC-UNSE-CONICET) equipped with a 532 nm laser excitation source with a total power of 18 mW. A single bacterial cell was brought into focus via a 100× objective. Each spectrum consisted of an average of 8 measurements per strain from at least 5 different colonies with an exposure time of 30 s in the range 100–2000 cm^−1^. The Raman Processing Software was used to import and analyze the data. To reduce variation and make fair comparisons, the mean spectra were normalized on the basis of the most intense peak appearing near 980 cm^−1^.

### 2.4. Cell Morphology of a Lactic Acid Bacteria Exposed to Bile Acids by Scanning Electron Microscopy

The effect of different BAs on the cell surface and morphology of *L. parabuchneri* CB12 was evaluated via scanning electron microscopy, using equipment and protocols developed at the Centro Integral de Microscopía Electrónica (CIME-UNT-CONICET). Briefly, the CB12 strain was grown at 37 °C for 16 h in the presence and absence of 0.25 mM CA, 2 mM DCA, or 7.5 mM TDCA. These concentrations were selected to achieve a survival rate of approximately 80%. To avoid artefact formation, cells were collected via centrifugation at low speed (600 g at 5 °C for 10 min), washed twice with 20 mM sodium phosphate buffer (pH 7.2), resuspended in the same volume of the buffer, fixed with Karnovsky’s fixative (1.66% glutaraldehyde, 2.66% p-formaldehyde in phosphate buffer), and stored at 4 °C until use.

### 2.5. Variations in the Zeta Potential of a Lactic Acid Bacteria Exposed to Bile Acids

*L. parabuchneri* CB12 was grown for 16 h at 37 °C in MRS broth, and cell suspensions were obtained in the same way as above. The A_560nm_ was adjusted by 0.4 ± 0.05 in 20 mM sodium phosphate buffer (pH 7.2), previously filtered through a 0.2 μm filter (Millipore). Increasing concentrations of TDCA (0.5 to 7 mM), CA (0.05 to 0.5 mM), or DCA (0.1 to 2 mM) were added to each cell suspension and allowed to stand for 5 min at room temperature and then placed in a DTS1070 cell (Malvern) using a disposable syringe.

Electrophoretic mobility was measured on a HORIBA SZ-100 nanoparticle analyzer at room temperature and at 40 V DC. Each sample was analyzed at least in triplicate.

## 3. Results and Discussion

### 3.1. Effect of Bile Acids on the Viability of Lactic Acid Strains

**Figure 1:**
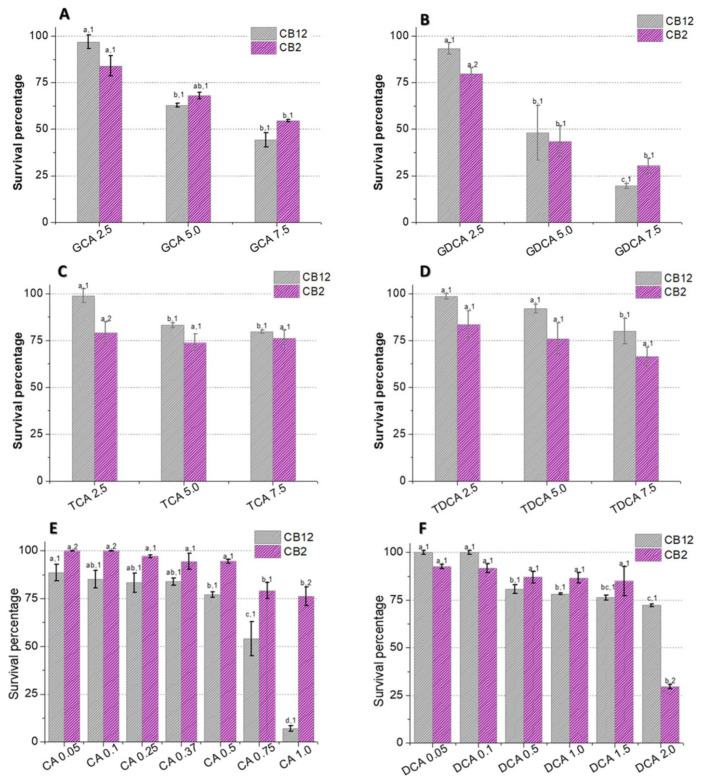
Survival of *L. parabuchneri* CB12 and *L. plantarum* CB2 strains in MRS medium with different concentrations of (**A**) GDCA (glycodeoxycholic acid), (**B**) GCA (glycocholic acid), (**C**) TDCA (taurodeoxycholic acid), (**D**) TCA (taurocholic acid), (**E**) DCA (deoxycholic acid), and (**F**) CA (cholic acid). To compare the effect of BAs on each strain and between strains, statistical analysis was carried out with the Minitab^®^ 19.1 software program. For the multiple comparisons test, Fisher’s LSD test was performed with a confidence level of 95%. Identical letters for each treatment indicate no statistically significant differences from the control. Equal numbers indicate no significant differences between strains.

The survival of *L. parabuchneri* CB12 and *L. plantarum* CB2 in the presence of different concentrations of BAs was evaluated (Figure 1). Our results showed that the effect of BAs on viability depends on the concentration and type of BA used, as well as on the strain. Among the conjugated species, glycoconjugates affected strain growth more than tauroconjugates. Furthermore, the greatest effect was observed with GDCA compared to GCA (*p*-value < 0.05), suggesting that not only do amino acid conjugates modify the BA–bacteria interaction; the steroid core is also involved, as other authors have pointed out [6]. 

As shown in Figure 1B, exposure to 2.5 mM GDCA resulted in a 7% and 20% reduction in cell viability of the CB12 and CB2 strains, respectively. Meanwhile, when 5 mM GDCA was added, the survival was approximately 45%, and at 7.5 mM, it was less than 30% for both strains. On the other hand, at 5 mM GCA, survival was 63 and 68%, while at 7.5 mM, it dropped to 44 y 54% for *L. parabuchneri* CB12 and *L. plantarum* CB2, respectively, but without significant differences in both cases (*p*-value > 0.05) (Figure 1A).

In the presence of TDCA (Figure 1D), a stepwise reduction in survival was observed in both strains, which showed 70 and 80% survival for CB2 and CB12, respectively, with the maximum concentration tested (7.5 mM). Similar behavior was observed with TCA, but the reduction in viability was less pronounced (Figure 1C). Indeed, for CB12, there are significant differences between concentrations of 2.5 mM (99% survival) and 5 mM (83% survival) but not at 7.5 mM (80% survival) (*p*-value < 0.05). For CB2, no significant differences were observed between treatments, but less survival was observed in relation to the CB12 strain (*p*-value < 0.05).

Free BAs, DCA (product of TDCA and GDCA hydrolysis), and CA (product of TCA and GCA hydrolysis) significantly affected the survival of both strains, with CA being the most deleterious (Figure 1E,F). The CB12 strain showed no significant changes in the presence of the lowest concentrations of DCA (0.05 and 0.1 mM), maintaining 100% survival. In contrast, *L. plantarum* CB2 decreased its survival by almost 10%. At 2 mM concentrations, 72 and 30% survival were observed for CB2 and CB12, respectively (Figure 1F). In the presence of CA, both strains show a gradual decrease in viability to values of 80% in the CB2 strain with the addition of 1 mM CA. Remarkably, the viability of the CB12 strain at the same concentration was less than 5% (Figure 1E).

In line with our results, Foley et al. (2021) [19] report higher toxicity of free BAs relative to their conjugated counterparts. However, among the free BAs, some authors found that DCA is more inhibitory than CA [5,20], while our results show an opposite trend. In this regard, Kurdi et al. (2006) [20] observed an approximately 10-fold difference between the MIC of CA and DCA. The authors attribute these findings to differences in the hydrophobicity properties of the two compounds since DCA possesses two OH groups, whereas CA possesses three. Indeed, the movement of a lipid molecule from one monolayer to the other (flip-flop) in unilamellar vesicles has been reported to be at least 10 times faster for DCA, implying that 10 times more DCA molecules can cross the membrane relative to CA in a specific time. On the other hand, a recent study by Foley et al. (2021) [19] shows that in *L. gasseri* and *L. acidophilus*, the MIC for DCA was twice as high as for CA, showing the higher toxicity of the latter. Furthermore, they found no correlation between the critical micellar concentration of the BAs and their MIC. Taken together, these findings suggest that the effect of BAs in LAB is not limited to the physicochemical properties of the molecules. Indeed, the response of LAB to BA depends on both the presence of intrinsic resistance mechanisms involving metabolic structures and pathways, as well as adaptive mechanisms involving genotypic and phenotypic modifications that arise as a consequence of the exposure of cells to stress in order to survive in its presence [15,16]. 

Based on the results obtained, we selected *L. parabuchneri* CB12 to continue our trials. 

### 3.2. Raman Analysis and Band Assignments of the CB12 Strain

**Figure 2:**
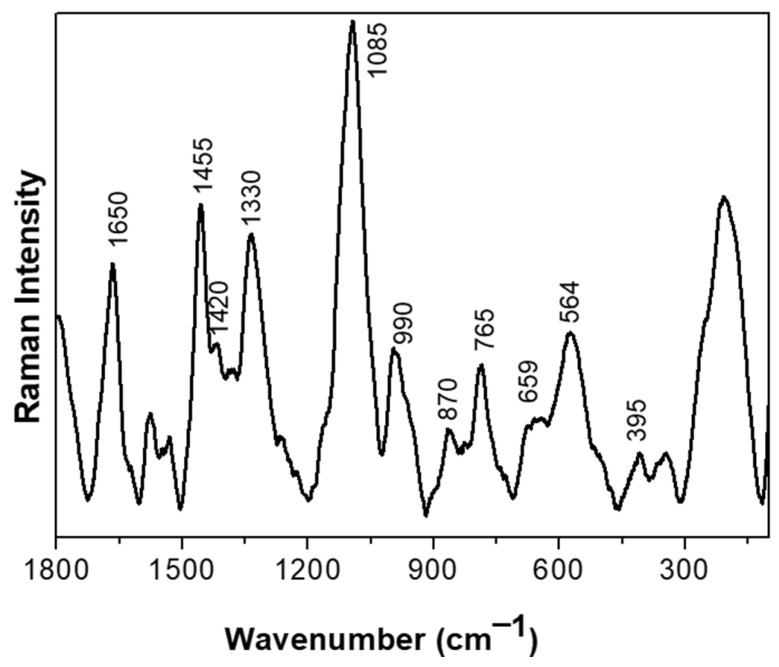
Raman spectra of the *L. parabuchneri* CB12 strain in the 1800–140 cm^−1^ region. Each spectrum resulted from the averaging of at least five spectra.

We set out to investigate the molecular aspects of the adaptive response to BAs of the CB12 strain using RAMAN spectroscopy. Figure 2 shows the normalized Raman spectra of the *L. parabuchneri* CB12 strain in the 1800–140 cm^−1^ region. Carbohydrates signals are attributed to band localized at 990 cm^−1^. For proteins, several bands were assigned; the amide I band was detected at ca. 1650 [21], as well as at 870, 659, and 564 cm^−1^, associated with C-N-C deformation, N-H deformation, and the disulfide S-S- vibration of the protein, respectively [22]. The vibration for the C-H deformation of lipids was detected at ca. 1420 cm^−1^, while the rocking mode vibration of CH_2_ of lipids was attributed to the band localized at 765 cm^−1^. Also, the band at 395 cm^−1^ was attributed to the phosphate groups of lipids’ bacterial component. DNA and RNA are detected at ca. 1,300 cm^−1^. The band localized at 1085 cm^−1^ was attributed to the C-N and C-C stretching vibration of nucleic acids, and the 1330 cm^−1^ band was attributed to the guanine and adenine signals of the DNA/RNA basis [23]. Bands for phospholipids were found in the 1500 to 1200 cm^−1^ range [24].

#### Effect of Bile Acids on the Main Cellular Macromolecules of the CB12 Strain

The effect of BAs on LAB is complex and multifactorial. Our results show that the addition of different concentrations of TDCA, DCA, or CA produced significant changes in bands associated with all cellular macromolecules, and the intensity of the changes was proportional to the concentration and type of BA used. 

**Figure 3:**
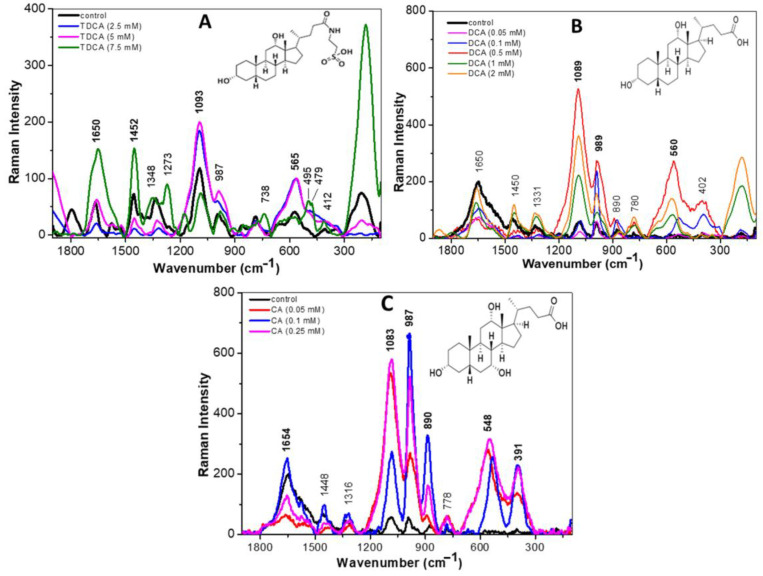
Raman spectra of the *L. parabuchneri* CB12 strain exposed to BA in the 1800–140 cm^−1^ region: (**A**) *L. parabuchneri* CB12 treated with different concentrations of TDCA (taurodeoxycholic acid); (**B**) *L. parabuchneri* CB12 treated with different concentrations of DCA (deoxycholic acid); (**C**) *L. parabuchneri* CB12 treated with different concentrations of CA (cholic acid).

Figure 3A shows the Raman spectra recorded for the CB12 strain grown in MRS broth (control—black line) and with TDCA at different concentrations. At low concentrations of TDCA (2.5 and 5 mM), an increase in the intensity of the bands at 1093, 987, and 565 cm^−1^, attributed to DNA, carbohydrates, and protein disulphide bridges, respectively, was observed. Also, a decrease in the intensity of 1650, 1452, and 394 cm^−1^ bands was observed, indicating a cleavage of the amide I bands and the lipid and phosphate groups, respectively. This would indicate a disruption of the cell membrane. At higher TDCA concentrations (7.5 and 10 mM), the effect was intensified since an increase in the signal of the mentioned bands was observed. From these changes it can be inferred that the presence of TDCA causes the breakdown and release of proteins, lipids, and polysaccharides, allowing them to vibrate more freely.

Figure 3B shows the Raman spectra of the CB12 strain in MRS broth (control—black line) and with DCA at different concentrations. In the presence of 0.05 mM DCA, no significant changes were observed compared to the control spectrum, which coincides with what was observed in the viability assays. With 0.1 mM DCA, an increase in the intensity of the band located at 989 cm^−1^, attributed to the C-O and C-C stretching modes of carbohydrates, was recorded. A similar behavior was observed for the bands located at 530 and 395 cm^−1^, attributed to the disulphide bonds of proteins and deformations to the phosphate group of lipids, respectively. At higher DCA concentrations (0.5, 1, and 2 mM), the modifications on the spectral profiles are much more evident with a shifted-to-higher wavenumber.

In the case of CA, with the 0.05 mM treatment, large changes were already observed with respect to the control condition, which would explain the greater inhibitory effect of this BA on the strain (Figure 3C). In particular, increases in the bands for DNA (1083 cm^−1^), carbohydrates (987 cm^−1^), and a slight increase in the protein signal due to C-N-C binding (890 cm^−1^) were observed. At higher concentrations (0.1 and 0.25 mM), the intensity of the bands attributed to the amide I bond of proteins (1654 cm^−1^), DNA (1083 cm^−1^), carbohydrates (987 cm^−1^), and phospholipids (391 cm^−1^) increased. Also, in a similar way as with DCA, a shift from the 522 cm^−1^ band towards the 548 cm^−1^ band was recorded. These changes were attributed to the oxidation of disulphide bridges and were proportional to the concentrations used. The lipid C-H bond (1426 cm^−1^) shifted ~6 cm^−1^ towards higher wavenumbers in the Raman spectrum of the CB12 in the presence of 0.25 and 0.5 mM CA. In the region below 1000 cm^−1^, the protein Raman signal located at 874 cm^−1^ (control) shifted towards higher wavenumbers (890 cm^−1^). Thus, it is evident that CA generates greater changes in the cellular component of CB12 when compared to TDCA and DCA, which may explain the higher sensitivity of the strain to this compound.

Due to their amphipathic character, BAs interact with, and incorporate into, the lipid bilayer of cells, causing changes in membrane integrity and interfering with the normal arrangement of molecules [5,20]. In this regard, our results show that exposure to conjugated and free BAs produced an increase in the intensity of the 395 cm^−1^ and 979 cm^−1^ bands, attributed to phospholipid and polysaccharide groups, respectively. In the case of CA, the effect was evident from the lowest concentration (0.25 mM), while higher concentrations of DCA and TDCA were needed to achieve the same effect.

Also, in all treatments, an increase in the intensity of the bands attributed to the amide I bond of the proteins (1654 cm^−1^) was observed, which was proportional to the concentration of each BA. In the same line, recent studies carried out by our group show that TDCA and DCA cause structural and functional changes in proteins, the effect being more evident in the presence of free BAs [25]. Moreover, a spectral shift in the band at 522 cm^−1^ observed in the control condition towards 530 cm^−1^ indicates a breakdown of the disulphide bridges when the cells were incubated with DCA and CA. In this regard, Cremers et al. (2014) [26] report that after exposure to BA, the bacterial cytosol becomes highly oxidative, indicating disulfide stress and oxidative stress. Remarkably, the Hsp33 chaperone is expressed in BAs-tolerant bacteria and is activated by the oxidation of four conserved cysteines to mitigate BAs-mediated protein aggregation effectively [26]. Finally, it was observed that the different BAs altered the band attributed to DNA in a concentration-dependent manner. 

These findings are consistent with previous results showing that bile exposure causes the overexpression of genes and proteins involved in the maturation of new proteins, refolding, or the degradation of denatured proteins, as well as DNA repair [15,27,28]. 

### 3.3. Effect of Bile Acids on Bacterial Surface of the CB12 Strain

**Figure 4:**
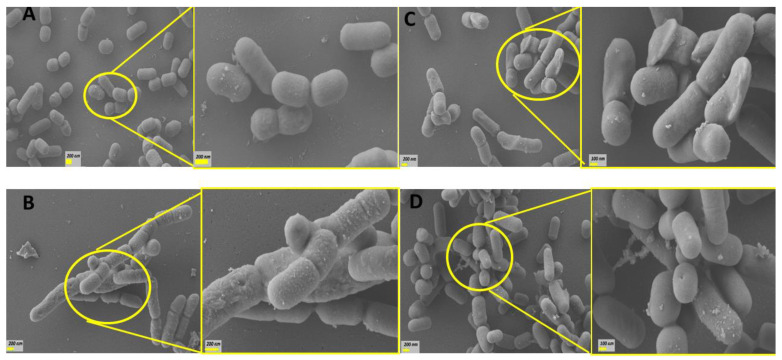
SEM images of the *L. parabuchneri* CB12 strain: (**A**) *L. parabuchneri* CB12 control; (**B**) *L. parabuchneri* CB12 treated with 7.5 mM of TDCA (taurodeoxycholic acid); (**C**) *L. parabuchneri* CB12 treated with 2 mM of DCA (deoxycholic acid); (**D**) CB12 treated with 7.5 mM of CA (cholic acid). On the left, the images show 10,000× magnification; and on the right, the images show 25,000× magnification.

Using scanning electron microscopy, the morphological changes of the CB12 strain exposed to different BAs was characterized. Concentrations were selected to achieve 80% viability based on our previous results (Figure 1). Figure 4A shows that untreated bacterial cells exhibit a characteristic, well-defined short rod shape and a smooth surface. The estimated size of 580 ± 50 in width and 1000 ± 200 nm in length is very close to that reported by Gumustop and Ortakcı (2022) [29] for *Lentilactobacillus parabuchneri* strains. No bacterial aggregates were observed.

Figure 4B shows that treatment with 7.5 mM TDCA caused varying degrees of damage to the surface of *L. parabuchneri* CB12. In particular, the surface appeared rougher and irregular with some depressions. An abundant amount of granular precipitate corresponding to the DCA released by the action of the BSH enzyme [17] was observed. Remarkably, elongated cells and incomplete septa were also evident, indicating that the presence of this BA delayed or avoided cell division of bacteria (Figure 4B right). In this regard, it was reported that exposure to bile induced the repression of genes involved in cell division, especially the tubulin analogue FtsZ, a finding that has also been described in bacteria subjected to other types of stress [30]. Additionally, it has been reported that bile stress dissipated the proton motive force leading to a decrease in the electrochemical energy necessary for cell growth and division, as well as other cellular processes such as stress resistance [15,20,31].

Treatment with free BAs such as DCA (Figure 4C) and CA (Figure 4D) caused changes of greater magnitude at lower concentrations. Thus, in bacterial cells treated with 2 mM DCA and 0.05 mM CA, significant surface distortions were observed with the presence of depressions and fold formation on the surface of many bacteria. In general, cells were longer than control cells (1083 and 1711 nm for CA and DCA, on average, respectively), and interrupted septa, indicative of incomplete cell division, were also observed. SEM micrographs showed bacterial aggregates, which would be related to modifications in their surface properties by exposure to BAs. Remarkably, exposure to CA revealed pore-like depressions that could result in the release of essential cellular materials. These findings could explain, in part, the higher sensitivity found in this strain to CA.

Previous studies showed that treatment of *Limosilactobacillus reuteri* CRL 1098 cells for 20 min with 5 mM DCA, examined via transmission electron microscopy, showed alterations in cell surface structure, such as pocked granules and low-electron-density vesicles. In contrast, 5 mM TDCA-treated cells showed no changes compared to the untreated control [32]. Also, using SEM, severe morphological defects were observed in *Staphylococcus (S.) aureus* exposed to CA and DCA compared to GCA and TCA. Indeed, cells treated with free BAs showed a shrunken, jagged appearance, with vesicles on their surface. These defects were less pronounced with conjugated BAs, demonstrating that these compounds possess less-potent antibacterial action on *S. aureus* than their deconjugated counterparts [33].

### 3.4. Effect of Bile Acids on Zeta Potential of the CB12 Strain

**Figure 5:**
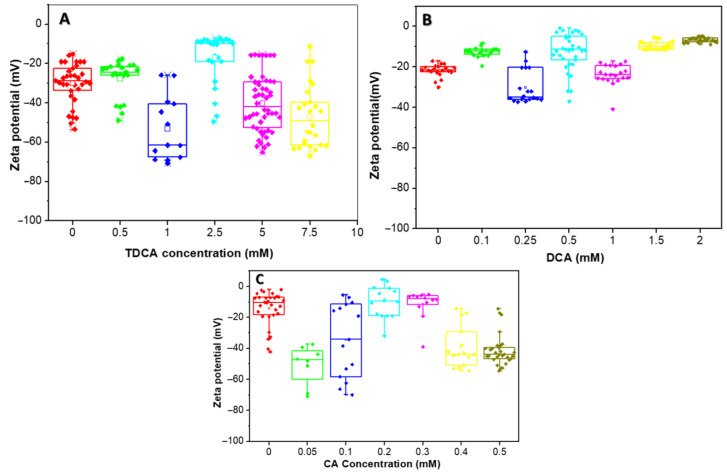
Zeta potential distribution of the *L. parabuchneri* CB12 strain as a function of BAs concentration: (**A**) TDCA (taurodeoxycholic acid); (**B**) DCA (deoxycholic acid); (**C**) CA (cholic acid). The box plot shows the zeta potential distribution for the different concentrations which are showed in different colors.

The effect of increasing concentrations of BAs on the ZP of *L. parabuchneri* CB12 under no-growth conditions is shown in Figure 5. The zeta potential distribution was plotted using a box plot. 

The zeta potential of the strain in the absence of TDCA was found to be extremely variable, with mean values around −30 mV (Figure 5A). These variations in zeta potential could be attributed to the different bacterial populations present in stationary phase, where there is an equilibrium between the number of new cells and the number of dying cells. The negative zeta potential is determined by both carboxylates (from lipopolysaccharides and proteins) and phosphates (from phospholipids, lipopolysaccharides, and proteins), among others.

In general, with the addition of increasing concentrations of TDCA, the ZP became more negative, reaching values close to −70 mV at 1 mM. However, when 2.5 mM TDCA was added, a population of bacteria with more-positive ZP values was observed, while another group maintained values close to −50 mV.

In the presence of DCA, small variations in ZP were observed, with a trend towards more positive values, except with 0.25 mM, where most cells reached values close to −40 mV (Figure 5B). Thus, with the addition of 0.1 mM DCA the ZP increased to an average value of −12 mV, while an opposite trend was observed at a concentration of 0.25 mM, where the average value was −35 mV. For higher concentrations of this BA, i.e., 1, 1.5, and 2 mM, an increase in the ZP was observed, with average values of −24 mV, −10 mV, and −6.6 mV, respectively.

Figure 5C shows the variation of ZP with CA concentration. For this BA, a similar behavior to TDCA was observed, except for 0.2 and 0.3 mM, whose values increased to about −8 mV, with little variability between data. Indeed, when 0.05 and 0.1 mM CA were used, the average zeta potential values were −49 and −34 mV, respectively, but with wide variability of the data, suggesting heterogeneous cell populations. For 0.4 and 0.5 mM concentrations, an average potential value around −40 mV was observed. 

The quantitative interpretation of the ZP is complex in simple systems and even more in microorganisms, where the charges of the system are not localized on a well-defined surface but are distributed throughout the cell wall and the plasma membrane [34].

**Figure 6:**
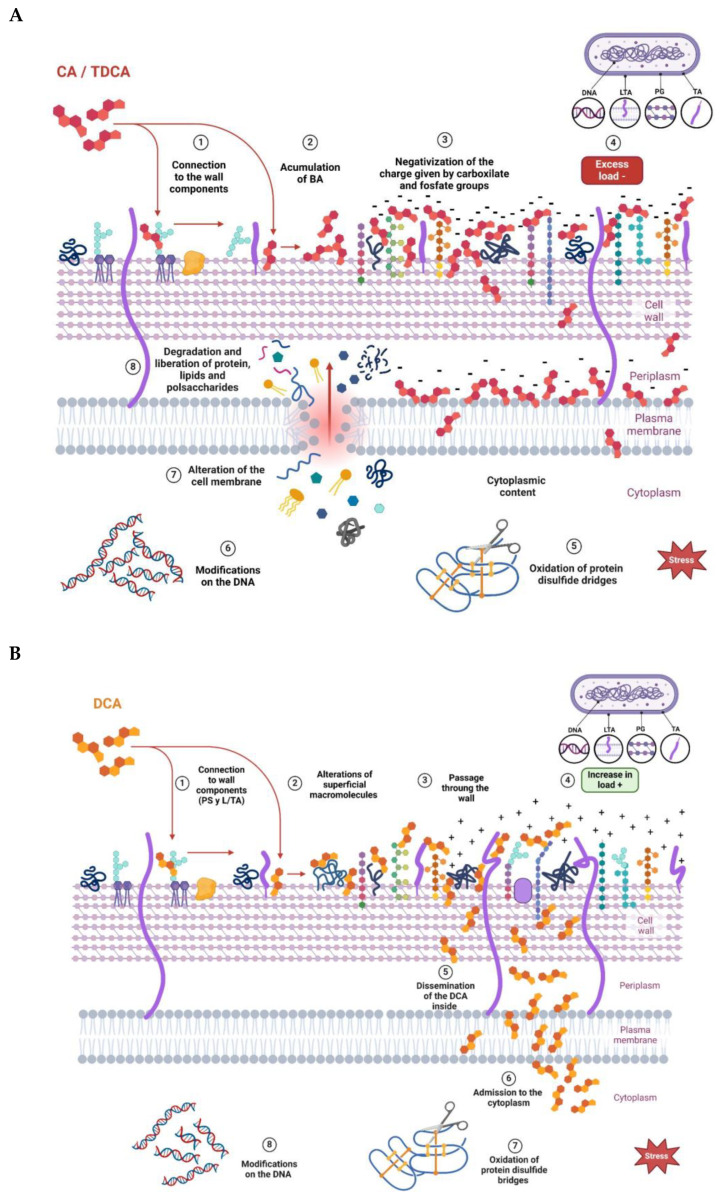
Proposed models to explain the effect of bile acids on the *L. parabuchneri* CB12 strain: (**A**) CA (cholic acid) and TDCA (taurodeoxycholic acid); (**B**) DCA (deoxycholic acid).

From the results obtained, it can be inferred that the three BAs evaluated produced changes in the net surface charge of the bacteria, with some differences among them. Figure 6 proposes a model with which to explain the interaction of the different BAs on the cell wall of a Gram-positive bacterium. At the beginning, the interaction occurs with different cell envelope components, such as peptidoglycan and lipoteichoic acid of the cell walls and phospholipids of the bacterial membrane, which is in agreement with results obtained via Raman microscopy. Once inside the cell, BAs denature intracellular proteins and nucleic acids. The decrease in ZP values observed at low concentrations of CA and TDCA can be explained as a phenomenon of incorporation and accumulation of BAs in the bacterial cell membrane (Figure 6A). It is important to note that TDCA was evaluated at higher concentrations than free BA. In this sense, in bifidobacteria, exposure to bile causes increased hydrophobicity and reduced ZP due to the accumulation of bile in the membranes [28]. On the other hand, DCA, at the concentrations tested, altered the ZP of the bacteria the least, with slight shifts towards more positive values (Figure 6B). This suggests that the compound diffuses rapidly into the cell compared to CA and TDCA. In this regard, Kurdi et al. (2006) [20] reported that due to its hydrophobicity, 10 times more DCA molecules can cross the membrane relative to CA in a given time. In the case of TDCA, the presence of the amino acid taurine provides increased hydrophilicity to the molecule. In addition, the TDCA molecule has a more negative surface potential and a higher dipole moment than DCA, according to studies by Bustos et al. (2022) [25] based on theoretical calculations. Moreover, at the pH of the tests (7.2), the weak acidic groups of both the cell wall polysaccharide and surface proteins may be dissociated, and exposure to BAs may denature the molecules and expose charged groups.

## 4. Conclusions

Bile acids are the main endogenous modulators of the composition and metabolic activity of the intestinal microbiota. Reciprocally, the intestinal microbiota extensively modifies the BAs pool through various enzymatic reactions.

Therefore, in the present work, the effect of BAs on the survival, surface properties, and main biological molecules of LAB was evaluated, with the purpose of deepening the knowledge of this interaction.

Our results showed that the effect of BAs on viability depended on the concentration and type of BAs used, as well as on the strain. Among the BAs conjugates, glycoconjugates showed a greater effect than tauroconjugates. Surprisingly, in both strains, CA showed significantly higher inhibitory activity than DCA but with significant differences between strains. This suggests that the effect depends on the structure of the BAs, the membrane-wall composition of the cells, and also on the stress-coping strategies of each strain. In general, *L. parabuchneri* CB12 showed high resistance to BAs, being sensitive only to high concentrations of CA, which makes it a good candidate for the design of functional probiotic supplements. Therefore, this strain was chosen as a model for Raman spectroscopy, SEM, and zeta potential studies in the presence of BAs. 

The Raman studies showed alterations at the protein, lipid, carbohydrate, and DNA levels. The magnitude of the damage depended on both the concentration and the BA used. The greatest effects were observed with CA, even when used at low concentrations, which is related to its greater inhibitory effect. A model with which to explain our main findings is proposed in Figure 6. In particular, our results show that exposure to conjugated and free BAs produced an increase in the intensity of the bands attributed to phospholipid and polysaccharide groups and to the amide I bond, as well as a disruption of disulfide bridges. Also, at high concentrations, an alteration of the band attributed to DNA was observed. 

Raman spectroscopy proved to be a valuable tool with which to evaluate global changes in macromolecules in a LAB exposed to BAs and allowed for a better understanding of this complex effect. To our knowledge, this is the first time that this technique has been used to evaluate the effect of BAs on bacteria. 

Moreover, the results obtained via SEM show that BAs cause surface distortions with the presence of depressions and fold formation on the surface of many bacteria. Interrupted septa, indicative of incomplete cell division, were also observed. 

It was observed that DCA, at the concentrations tested, was the one that least altered the ZP of the bacteria, with gradual changes towards more positive values. This may suggest that the compound diffuses rapidly into the cell (Figure 6B). In contrast, CA and TDCA, at low concentrations, decrease ZP, which can be interpreted as a phenomenon of adsorption and accumulation of the BAs on the membrane wall of the bacterium prior to their entry (Figure 6A). This could explain, at least in part, why CA at lower concentrations causes a significant effect on membranes and, consequently, on viability. 

This work provides solid evidence on the effects of BAs on LAB and allows us to deepen our knowledge of new strains that could be used to modulate the composition of the intestinal microbiota.

## Data Availability

The data that support the findings of this study are available on request from the corresponding author.
